# A Dictionary of Practical Medicine; Comprising General Pathology, the Nature and Treatment of Diseases, &c.

**Published:** 1837-07

**Authors:** 


Art. VIII.—*A Dictionary of Practical Medicine; comprising general Pathology, the Nature and Treatment of Diseases, &c.* By James Copland, m.d. f.r.s. &c. Part IV.—*London*, 1837. 8vo. pp. 304.

As the first two parts of this Dictionary were published before we commenced our labours in this Journal, and as the remaining two parts are promised before long, we shall defer our notice of the work until it is completed. We cannot, however, allow this opportunity to pass without bearing testimony to the extraordinary merits of the publication, and recommending it, in the strongest terms, to the attention of our readers. Considered as the production of an individual, this work is one of the most extraordinary that has ever appeared, for its size, comprehensiveness, accuracy, and learning; and, although necessarily inferior in certain respects, from its very plan, to some works of a like kind, the composition of a large body of writers associated for the purpose, it is superior to these in the general unity of the principles and practice laid down in it, and assuredly excels them all in depth and variety of research. For a practical work, it is, indeed, almost too learned; as the student is liable to be perplexed by the extreme variety of views and opinions cited from the writers of all times and countries; but this, if a fault, is certainly one on the right side, and one which we should be glad to have more grounds for complaining of in some other works of a like kind.

The present part contains the subjects from *Fever* (Remittent) to *Heart* (Hypertrophy of), inclusive. We sincerely hope that the learned and indefatigable author may be enabled to complete his undertaking within the time stated; although we are too well acquainted with the nature and extent of his labours to blame him if this should not be the case. At any rate, whenever complete, this work must remain an imperishable monument of his talents, learning, and industry.

